# Herbal Medicines: Malaysian Women's Knowledge and Practice

**DOI:** 10.1155/2013/438139

**Published:** 2013-09-05

**Authors:** Law Kim Sooi, Soon Lean Keng

**Affiliations:** ^1^Advanced Medical & Dental Institute, Universiti Sains Malaysia, 13200 Kepala Batas, Penang, Malaysia; ^2^School of Health Sciences, Universiti Sains Malaysia, 16150 Kelantan, Malaysia

## Abstract

This is a cross-sectional, descriptive study among Malay women admitted in the antenatal and postnatal ward to determine the prevalence and use of herbal medicines during pregnancy and elemental analysis in the most popular herbs. A total of 460 women were surveyed. Herbal medicine use during pregnancy was 34.3%, while 73% utilized herbal medicines during labor, because of a belief that it may shorten and ease labor. The most commonly used herbal medicines in pregnancy were *Anastatica hierochuntica* L. (60.1%) followed by coconut oil (35.4%). The majority of women (89.2%) used only one type of herbal medicines and took one capsule/glass (38%) per day. Herbal medicines use by pregnant women is largely unsupervised (81%), with most women getting information from their parents (60.7%) and buying the products directly from traditional midwives (32.2%) and 77% agreed upon its efficacy and safety. From the 460 respondents, 89.8% women were in the low end of the herbs knowledge. There was a significant difference found between knowledge score and income (*P* < 0.05). Microdiffraction analysis revealed significant presence of carbon, oxygen, silica, calcium, magnesium, aluminium, potassium, zinc, and iron that were found in *Anastatica hierochuntica* L. and proved to have good benefits for pregnancy.

## 1. Introduction

 Traditional medicine or complementary and alternative medicine (TM/CAM) is an integral part of the practice of ancient civilization to improve the health and well-being of people in countries such as India, China, Malaysia, and throughout the world [1]. The various modalities currently practiced are based on theory, beliefs, and experiences that are indigenous to the different cultures and developed and handed down from generation to generation [2]. 

Herbal medicine usage is not only popular among individuals, but also among primary health care providers in developing countries.   Herbal medicines are also practiced in countries where conventional medicines are predominant [3]. The World Health Organization [4] reported that 70% to 80% of the world population relies mainly on herbal sources for their primary medicines. A study by Matthews et al. [5] found that herbal medicines were used in many countries to treat pregnancy related illness and to encourage healthy pregnancies and overall well-being. Previous studies indicated that although the practice of herbal consumption in pregnancy was very popular, they reported a lack of evidence for the safety and efficacy of herbal medicines utilized during pregnancy [6–9]. Similarly, other studies showed that although herbal medicines were unproven scientifically, 4% to 62% of pregnant women continued to consume herbal medicines over their pregnancy period without the lack of knowledge about its safety and benefit [5, 9–11].

Malaysia is a multicultural country that has rich traditional practice modalities. Among these modalities there is the use of herbal medicine for the treatment of various ailments during pregnancy [8, 9, 12]. Herbal medicines are becoming increasingly popular and are regarded as important to the public as well as to the scientific community [13]. The beliefs of the majority of ethnic groups in the Malaysian population is that herbal products do not contain harmful chemicals and are free of side effects when compared to commercially available pharmaceutical drugs [9, 14]. In Malaysia, the uses of herbal medicines are based on practical experiences, observations, and rituals derived from socioreligious beliefs passed down from one generation to another. These practices have been observed within the Malay communities and claim to be important for reasons of health and well-being, including being beneficial during pregnancy. Although various studies had been published on medications used during pregnancy, there is a lack of evidence for safety and efficacy on these herbal medicines during pregnancy [8–10, 14]. Since many women used herbal medicines during pregnancy, which might produce potential adverse side effects on the mother and foetus, such practice modalities should raise concerns among healthcare professionals and consumers on the issue of safety and efficacy [8–10, 15]. In addition, there are limited, and, in many cases, a lack of data to the extent of Malaysian women's use of herbs during pregnancy.

## 2. Objectives

This study was to determine the prevalence, to describe the pattern of herbal medicine usage during pregnancy among the Malay women in Kelantan, and to explore their knowledge and attitude of safety towards these herbal medicines. Because herbal medicines are known to have trace minerals, therefore elemental analysis was determined in the most popular herb using energy dispersive X-ray (EDX) microanalysis to identify relationships of these structures to the possible claimed effects which is the secondary aim of this research.

## 3. Materials and Methods

The present cross-sectional, descriptive study was conducted at the antenatal and postnatal ward in Hospital Universiti Sains Malaysia, Kelantan, Malaysia. The study consisted of women in their last trimester of pregnancy admitted as in-patients for delivery and within two days after childbirth at the postnatal ward. The survey involved interview data collection via prepared tested structured survey questionnaires. The questionnaires in this study consisted of four sections.

 Section A: demographic data of the respondents.
 Section B: the pattern of herbal medicines use during pregnancy, the timing of administration (trimesters 1, 2, and 3), their usual source of information, and beliefs about the safety and efficacy of traditional herbs.
 Section C: the types of herbal medicines use during pregnancy.
 Section D: the knowledge and attitude of safety towards the herbal medicines usage during pregnancy.

EDX was performed on the most popularselected herb commonly used based on the survey questionnaires.

### 3.1. Plant Material

*Anastatica hierochuntica *L. (Sanggul Fatimah) was obtained from the local market in Kota Bharu, Kelantan, Malaysia, and in Medina, Saudi Arabia.

### 3.2. Specimen Preparation

*Anastatica hierochuntica* L. dried branches, flowers, and grounded powder forms were used in this study and digital stereomicroscope images were taken. Each preparation was initially cyclically dehydrated through a series of ethanol washes (75%, 95%, and 100%) for 15 minutes each with 3 changes. Following, the preparations were then removed from the ethanol and dried via hexamethyl-disilazane evaporative technique for 10 minutes. The preparations were transferred into specimen plate and redried in a critical point dryer for about half an hour. Following the drying methods, the preparations were prepared for coating within the sputter coater.

### 3.3. Elemental Analysis

Elemental identification of the preparations was detected using Energy Dispersive X-Ray (EDX) (Oxford Inca EDX model 7353, England) attached to the variable pressure scanning electron microscope (VPSEM), operated at an accelerating voltage of 20 kV. Minimal three EDX spectrums were acquired for elemental characterization for each morphological feature identified.

### 3.4. Statistical Analysis

SPSS version 18.0 was used to analyze the data. Descriptive statistics was used to describe the characteristics and patterns of the traditional herbal medicine usage during pregnancy among the Kelantanese Malay. Categorical variables were presented as frequency and percentage. Continuous variables were presented as mean and standard deviation (SD). 

Inferential statistics was used to draw conclusions about the data and to compare groups or to explore relationships between the variables. Chi-square testing was used to identify the relationship between sociodemographic factors and herbal use among pregnant Malay women in Kelantan, Malaysia. An independent *t*-test was used to compare the mean knowledge score of the pregnant Malay women for sociodemographic factors with 2 groups of categorical variables, whereas analysis of variance (ANOVA) was to compare the mean knowledge score of these women for sociodemographic factors with more than 2 groups of categorical variables. At the 5% level of significance, all null hypothesis was rejected if *P* < 0.05.

Ethical approval for the study was gained from the relevant committees, the Human Research Ethics Committee, Universiti Sains Malaysia, and the Director of Hospital Universiti Sains Malaysia. The patients were informed about the study and were invited to participate. After obtaining informed consent, the study was conducted based on the structured questionnaire and all responses were documented.

## 4. Results

A total of 460 mothers responded in this study. The age of the mothers ranged between 18 years old to 46 years old. The mean age of the mothers was 31.3 years old. The sample was divided into four groups: below 20 years, 21–30 years, 31–40 years, and 41 years and above. The majority of the women (43%) were between the age of 31 and 40 years; 42.4% were between the age of 20 and 30 years; 11.1% were 41 years and above; 3.5% were younger than 20 years. A majority of the mothers were housewives (55.7%) and had attended formal education up to secondary school (61.1%). Most of the mothers had 2 to 5 children. Demographic characteristics of respondents are shown in Table 1.

158 (34.3%) women used at least one type of herbal medicine during pregnancy. A Chi-square test was used to analyze the association between demographic variables and herb usage. Age groups were combined into two groups women below 30 years and women above 30 years. The relationship between age and herbal use was found to be significant (*P* < 0.05). For convenience and analysis purposes, occupation was divided into two groups: unemployed and employed. 21% of the unemployed women were herbal users during pregnancy and 13.2% of the employed women reported using at least one herb during pregnancy. The relationship between occupation and herb usage was not significant (*P* > 0.05). 23.3% of the women who had a primary school education level reported using herbs during pregnancy; among the women with a secondary school education level, 3% reported using herbs; among those women with a tertiary education level, 8% reported use of herbs. The relationship between education level and herbal use was not significant (*P* > 0.05). 19.8% of women from income less than RM1000 reported using herbs; and 11.3% from income RM1000–RM3000 and 3.2% from income more than RM3000 reported using herbs during pregnancy. The relationship between income and herbal use was found to be significant (*P* < 0.05). The relationship between sociodemographic characteristics and herbal use during pregnancy is shown in Table 2.

Use of herbal medicines according to the trimester of pregnancy was shown in Table 3. The majority of women (73.4%) used herbs during labor; 7.6% of the women had taken at least one herb during the first trimester. However, no herbs were used during the second trimester.

**Figure 1:**
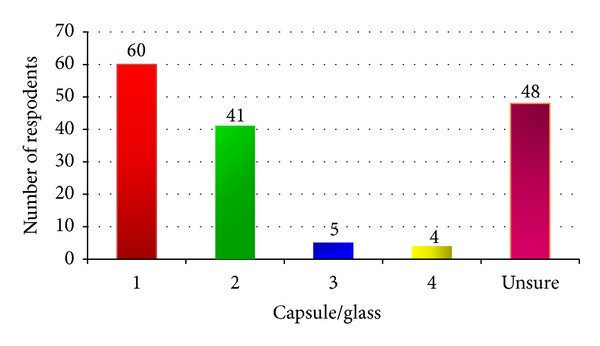
Amount of herbal medicines consumed daily.

Herbs were consumed in the form of a tea or capsule.  Figure 1 showed that the majority (60) (38%) of women took one capsule or drank one glass of herbal tea per day and 48 (30.4%) women were unsure of the amount they consumed daily.

From the results, 12.7% of the pregnant women had taken herbal medicines with conventional medicines on the same day.  Table 4 shows the information sources about herbal medicines. The information sources for a majority (60.8%) of the women during pregnancy were from their parents, followed by traditional midwives (10.1%), friends (10.1%), relatives (9.5%), parents-in-law (5.1%), mass media (1.9%), company (1.9%), and healthcare provider (0.6%).

In this study, it was found that women used herbal medicines during pregnancy for a variety of reasons. The most commonly reported reasons for the use of herbal medicines during pregnancy were to facilitate labor (89.2%). This is followed by a promotion of health status (31%), of traditional practice (22.8%) and to relieve common discomfort during pregnancy (10.8%), to keep warm (10.8%), to keep sexual pleasure (7%), to restore youth (7%), to prevent whitish discharge (6.3%), and to promote fetal physical health and intelligence (5.7%).  Table 5 shows common reasons for the use of herbal medicines during pregnancy.

**Figure 2:**
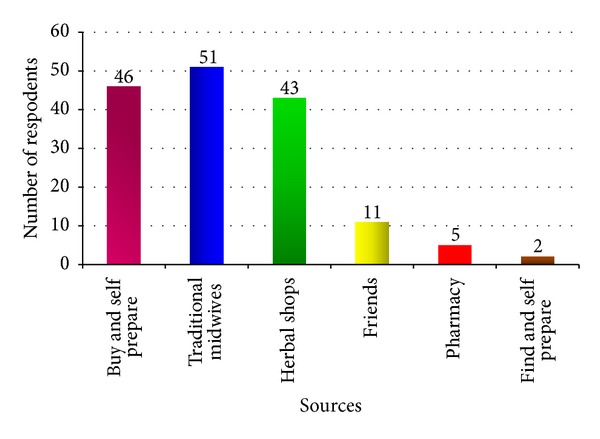
Sources of herbal medicines.

The results also showed that the majority (51) (32.2%) of women bought herbal medicines directly from traditional midwives followed by store purchases and self-preparations 46 (29.1%), or from herbal shops 43 (27.2%).  Figure 2 showed sources of herbal medicines.

77.2% of women perceived herbal medicines as being safe and effective because herbs are “natural” substances and do not contain any dangerous chemicals and also because the practice of using medicinal herbs has been going on for many generations. It is therefore considered safer than conventional medications during pregnancy. More than a half (51.9%) of the women found the herbal medicines to be effective. Only 10.1% of women felt that herbal medications were not effective 31% of women were unsure or did not notice any beneficial effects while taking herbal medications during pregnancy. Ten percent of the childbearing women strongly agreed and 73.3% agreed with the integration of traditional medicines/herbs with modern medications. The study also found that 81.6% of women did not report their use of herbal medication to their doctor. However, 91.8% of the women were more likely to tell their doctor about using herbal medications, when asked.

In this study, the most common herb used during pregnancy was Sanggul Fatimah (*Anastatica hierochuntica* L.) (63.9%), coconut oil (minyak selusuh) (33.5%), unidentified herbs prepared by traditional midwives (6.3%), homeopathy (11.4%), *Zingiber officinale *(3.2%), and others as shown in Table 6. Unidentified herbs prepared by traditional midwives were not listed in the questionnaire but were used by some women during pregnancy.

In this study, the mean score of knowledge was 14.34 ± 3.37 (mean ± SD). Out of 460 respondents, 89.8% of women were in the low-herb knowledge score and 10.2% of women were in the high-herb knowledge score. Among the women in the high score category, 42.6% had used herbal medicines during pregnancy.

No statistical significant difference was found between the knowledge score and sociodemographic characteristics of age, occupation, education level, and parity (*P* > 0.05). However, there was a significant difference found between knowledge score and income (*P* < 0.05) (Table 7).

Out of 460 respondents, only 8.5% of women said that they knew the ingredients of the herbal medications. Nevertheless, none of them could list what were the ingredients in the herbal medications they consumed. In total, 18.9% of the women claimed that products containing herbal medicines had added value or adulterants. However, only 11.5% of the women were aware that herbal medicines may be exposed to mercury, microbial, or contamination and 11.1% answered that they knew how the herbal medicines had been processed. Moreover, most women had a very limited knowledge in the procedure for preparing herbal medicines. Half of the respondents 49.3% said that herbal medicines used during pregnancy could cause abortion, fetal growth retardation (19.8%), premature delivery (19.1%), malformation (27%), and fetal death (20%). However, 27.6% of the respondents also believed that herbal medicines could increase the well-being of the mother and fetus (38.5%).

In this study, the weight percentages of elements were detected in *Anastatica hierochuntica *L. as shown in Table 8. Elemental analysis result showed that samples examined had useful minerals such as calcium, magnesium, aluminum, potassium, zinc, and iron, apart from the ubiquitous carbon, oxygen, and silica.

**Figure 3:**
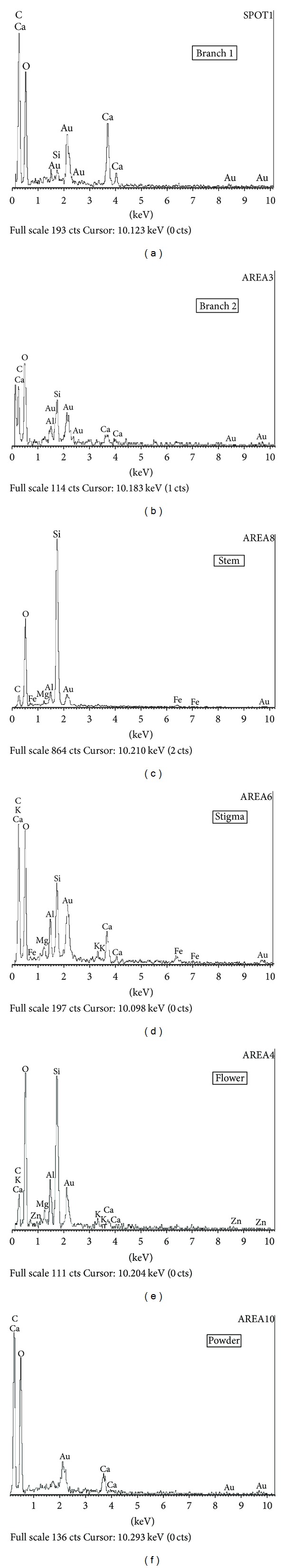
Energy Dispersive X-ray (EDX) for elemental analysis from the selected area of *Anastatica hierochuntica*. C: carbon; O: oxygen; Ca: calcium; Au: gold; Si: silicon; Al: aluminium; Mg: magnesium; K: potassium; Fe: iron; Zn: zinc.

This study presents the result of element composition of *Anastatica hierochuntica* L. using EDX technique. The EDX spectra obtained from the selected area are given in Figure 3 while their elemental compositions are listed in Tables 8 and 9.

As can be seen from Figure 3, branch 2 from *Anastatica hierochuntica *L. showed the presence of various elements such as O, C, Si, and Ca, in which O is the highest percentage (46.73%) followed by C while small quantities of Si and Ca were also detected (Tables 8 and 9).

O, C, Si, Ca, and Al were detected in the *Anastatica hierochuntica *L. from branch 1, and both O and C elements are more or less present in equal ratio. Small amounts of Si, Ca, and Al were also present in these extracts.

Similarly O, C, Mg, Al, Si, K, and Ca were detected in area of stem and stigma. O was still in the highest percentage followed by C, where these are present in almost 3 : 1 ratio. The rest of the detected elements were present in small quantities. Zn was detected only in the area of stem while Fe was found in the area of stigma only in small quantities.

The crude extracts derived from various parts (area flower and powder) of *Anastatica hierochuntica *L. showed some variations in their elemental constituents and composition as shown in the area of flower and powder. O, C, Mg, Al, Si, and Fe were detected in the area of flower while powder displayed the presence of O, C, and Ca only. 

As shown in Tables 8 and 9 and Figure 3, the percentage composition of O was found as the major element in all area followed by C. Both of these elements are more or less present in equal ratio except in area of stem and flower where these are present in almost 3 : 1 ratio. The rest of the detected elements were present in small quantities in all areas. Fe was detected only in the area of stigma and flower while Zn was found in the area of stem only. 

## 5. Discussion

Herbal medicine use by pregnant Malays in this present study was significantly high, and women indicated that they would continue to use it. The high prevalence of herbal medicines utilization may probably be due to the strong believes that these herbs are safe. This confirmed the findings from previous studies where many women strongly believed in safety of herbal medicines use in pregnancy and perceived absence of side effects [9, 12, 16]. Therefore, knowledge of its use will continue to be passed down from generation to generations. This indicates a need for more evidence-based research related to the safety of herbal medicines, since inappropriate dosage and dosage forms were noted in the study. These findings suggest that the inappropriateness of dosage and dosage forms may be probably due to different cultural beliefs and practices in Malaysia [8, 9, 12, 14, 16]. This confirmed the findings from a previous study where women from different geopolitical zones cited diverse views on beliefs and effects in their consumption of herbal medicines during pregnancy [17]. 

Most respondents in the present study believed that herbal remedies facilitate labor. This finding was in support of data from Azriani et al.'s (2008) study in Tumpat, Kelantan, Malaysia, among 210 mothers which indicated that 108 mothers (51.4%) used at least one type of herbal medicines during pregnancy and the most common indication was to facilitate labor (89.8%) [12]. In our study, *Anastatica hierochuntica *L. was the most commonly used herbal medicine taken during pregnancy. This was quite expected since a substantial number of respondents believed that *Anastatica hierochuntica *L. promotes faster delivery, thus suggesting that this herbal medicine has effects that caninduce and expedite labor and also open the woman's cervix [9]. 

The elemental analysis found that *Anastatica hierochuntica *L. has beneficial properties such as silica, magnesium, calcium, oxygen, potassium, iron, and zinc which play an important role in maintenance of human health. This might explain why these women consumed *Anastatica hierochuntica *L. during pregnancy. 

Because of the pharmacological active component in herbal medicines, there is a possibility of potential harm to the fetus. Caution should therefore be exercised with the use of herbal medicines during pregnancy [9, 12, 16]. It is therefore essential to determine the mineral elements in herbal medicines and to establish the levels of some metallic elements in commonly used herbal medicines because, at elevated levels, these metals could be dangerous and toxic [18]. Furthermore, health care providers need to be aware of the common herbal medicines consumed by pregnant women and need to be proactive with pregnant women who consider using herbal medicine. They also need to be cognizant with the evidence base study regarding dosage and dosage forms, as well as the potential benefits or harmful side effects of herbal medicines. Information obtained could provide benefits in treating or preventing illness, including relevant supporting evidence that is more conclusive in antenatal care and practices. Knowledge of the elemental content in herbal medicines is very important since many trace elements play significant roles in the formation of active constituents responsible for the curative properties in human [19]. Although there is little published information on contraindications between herbal medications and drugs or other herbs, health care professionals should at least be aware of the trends in herbal medicine utilization during pregnancy so that they may educate themselves on herbal modes of action and avoid potentially dangerous interactions in their patients.

Herbal preparations may be used to treat a variety of conditions, but the pregnant woman needs to be aware of the actions, benefits, and potential dangers associated with herbal medications. Thus, herbal practices during pregnancy need to be identified so that appropriate educational intervention may take place. Actual knowledge of herbs and their properties should be established by health care providers so that educational needs can be determined. Research should be conducted to evaluate actual knowledge of herbal practices. Once the use and knowledge of herbs has been established, investigations should be conducted to determine the effect of herbs upon the pregnant mothers and their unborn children. The incidence of herb usage in pregnant women who miscarried or delivered a nonviable infant needs to be evaluated in a further study [9, 12, 16].

*Anastatica hierochuntica *L. revealed inert significant presence of carbon (C), oxygen (O), silica (Si), calcium (Ca), magnesium (Mg), aluminum (Al), potassium (K), zinc (Zn), and iron (Fe). Some of the elements such as calcium, magnesium, zinc, and iron that were found in *Anastatica hierochuntica *L. are known to have good benefits for pregnancy. Calcium is essential for bone formation and growth as well as muscle and nerve regulation. During pregnancy, calcium is critical to the baby's developing skeletal system and teeth. Calcium also can reduce the risk of developing pregnancy-related high blood pressure [20]. Meanwhile, magnesium helps regulate energy metabolism, blood sugar levels, and nerve transmissions. It works with calcium to regulate muscle contractions, including those in the uterus [21].

## 6. Conclusion

Herbal medicines usage is still being held as an important practice among the Kelantanese Malay women. The usage of herbs during pregnancy has not been previously reported by the Kelantanese Malay women to their health care providers. Therefore, pregnancy care providers should be aware of the common herbal medicines used and the need to know with evidence-based research regarding potential benefits or harmful side effects of herbal medications. A detailed study is therefore needed to establish the efficacy and safety of these herbs to ensure the well-being of the mother and foetus. Many herbs are believed to be beneficial or helpful in easing certain pregnancy related problems but there is no scientific basis for that belief. The active ingredients of plant extracts are chemicals that are similar to those in purified medications and, therefore, they have the same potential to cause serious adverse effects. Obstetricians should advise women not to expose their foetuses to the risks of herbal medicines.

Documentation of knowledge regarding the use and elements found in common herbs are needed, as information gathered could help in educational intervention essential to the Malaysian healthcare system. This study provides the foundation for further research on the possible side effects of herbal medications. Healthcare professionals, particularly nurses and midwives in Malaysia, who provide care for antepartum women in the clinical setting, need to be cognizant of the herbal medical practices and educate the antepartum woman regarding the potential benefits and dangers of herbal medications on them and her unborn foetus.

## Figures and Tables

**Table 1:** Distribution of respondent by sociodemographic characteristics (*n* = 460).

Characteristic	*n*	%	Mean	Min/Max
			31.3	18/46
Age				
<20	16	3.5		
21–30	195	42.4		
31–40	198	43.0		
>41	51	11.1		
Occupation				
Housewife	256	55.7		
Supportive staff	114	24.8		
Own job	22	4.8		
Professional	37	8.0		
Others	31	6.7		
Education				
Non	16	3.5		
Primary	58	12.6		
Secondary	281	61.1		
Diploma	63	13.7		
Degree and above	42	9.1		
Income				
<RM1000	298	64.8		
RM1000–RM3000	130	28.3		
>RM3000	32	7.0		
Gravidity				
Primid.	102	22.2		
2–5	263	57.2		
6 and above	95	20.7		

**Table 2:** Relationship between sociodemographic characteristics and herbal medicines use during pregnancy.

Characteristic	Herbal users *n* (%)	Herbal nonusers *n* (%)	*X* ^2^ stat (df)	*P* value
Age				
<30	48 (10.4)	180 (39.1)		
>30	110 (23.9)	122 (26.6)	19.20	<0.001*
Occupation				
Unemployed	97 (21.1)	181 (39.4)		
Employed	61 (13.2)	121 (26.3)	4.89	0.087
Education				
Primary	107 (23.3)	190 (41.3)		
Secondary	14 (3.0)	44 (9.6)		
Tertiary	37 (8.0)	68 (14.8)	3.09	0.213
Income				
<RM1000	91 (19.8)	207 (45.0)		
RM1000–RM3000	52 (11.3)	78 (17.0)		
>RM3000	15 (3.2)	17 (3.7)	22.44	<0.001*
Gravidity				
Primid.	24 (5.2)	78 (17.0)		
2–5	91 (19.8)	172 (37.4)		
6 and above	43 (9.3)	52 (11.3)	10.32	0.006*

*P < 0.05.

**Table 3:** Use of herbal medicines according to the trimester of pregnancy.

Gestation period	*n*	% of total respondents
During 1st trimester	12	7.6
During 2nd trimester	0	0
During 3rd trimester	28	17.7
During entire pregnancy	2	1.3
During labor	116	73.4

**Table 4:** Information source of herbal medicines.

Information source	*n*	%
Parents	96	60.8
Traditional midwives	16	10.1
Friends	16	10.1
Relative	15	9.5
Parents-in-law	8	5.1
Mass media	3	1.9
Company	3	1.9
Healthcare providers	1	0.6

**Table 5:** Common reasons for herbal medicinal usage during pregnancy (*n* = 158).

Reasons	Yes *n* (%)	No *n* (%)
Facilitate labor	141 (89.2)	17 (10.8)
Promote health status	49 (31.0)	109 (69.0)
Traditional practice	36 (22.8)	122 (77.2)
Relieve common discomfort during pregnancy	17 (10.8)	141 (89.2)
Keep warm	17 (10.8)	141 (89.2)
Sexual pleasure	11 (7.0)	147 (93.0)
Restore youth	11 (7.0)	147 (93.0)
Prevent whitish discharge	10 (6.3)	148 (93.7)
Promote fetal physical health and intelligence	9 (5.7)	149 (94.3)

(Multiple responses were allowed).

**Table 6:** Most common herbal medicines use during the pregnancy period.

Types of herb	*n*	%
S. Fatimah (*Anastatica hierochuntica *L.)	101	63.9
Minyak Selusuh (Coconut Oil)	53	33.5
Unidentified Herbs	10	6.3
Halia (*Zingiber officinale*)	5	3.2
Bawang Merah (*Allium ascalonicum*)	5	3.2
Bawang Putih (*Allium sativum*)	4	2.5
Serai (*Cymbopogon citratus*)	4	2.5
Kunyit (*Curruma longa*)	2	1.2
Manjakani (*Croton caudatus*)	2	1.2
Inai (*Lawsonia inermis*)	2	1.2
Sirih (*Piper betle *L.)	2	1.2
Jarum Mas (*Striga asiatica*)	2	1.2
Pegaga (*Centella asiatica *L.)	1	0.6
Sepang (*Caesalpinia sappan*)	1	0.6
Homeopathy	18	11.4

**Table 7:** The comparison of mean knowledge score of herbal medicines and sociodemographic characteristics using independent *t*-test and ANOVA.

Sociodemographic characteristics	Mean (SD)	*t*-stat (df)	*P* value
Age			
<30	4.0 (2.2)	0.069	0.945
>30	4.0 (2.2)		
Occupation			
Unemployed	3.9 (2.3)	0.271	0.786
Employed	3.9 (2.1)		
Education			
Primary	3.5 (2.3)	2.230	0.109*
Secondary	3.9 (2.1)		
Tertiary	4.4 (2.1)		
Income			
<RM1000	3.7 (2.1)	4.349	<0.050*
RM1000–RM3000	4.3 (2.4)		
>RM3000	4.3 (2.2)		
Gravidity			
Primid	3.9 (2.1)	0.010	0.990*
2–5	3.9 (2.4)		
6 & above	3.9 (2.0)		
Prevalence of herbal medicine usage			
Yes	4.0 (2.3)	0.339	0.735
No	3.9 (2.2)		

*ANOVA.

**Table:** (a)

Branch 1			Stigma			Stem		
Element	Weight%	Atomic%	Element	Weight%	Atomic%	Element	Weight%	Atomic%
C K	38.78	48.13	C K	35.19	46.77	C K	16.98	24.81
O K	49.31	45.95	O K	42.73	42.63	O K	52.42	57.50
Al K	2.18	1.21	Mg K	0.85	0.56	Mg K	1.25	0.90
Si K	6.89	3.66	Al K	3.61	2.13	Al K	5.09	3.31
Ca K	2.84	1.05	Si K	7.19	4.09	Si K	18.51	11.57
			K K	1.19	0.49	K K	1.33	0.59
			Ca K	6.11	2.43	Ca K	0.78	0.34
			Fe K	3.14	0.90	Zn K	3.64	0.98
**Totals**	**100.00**		**Totals**	**100.00**		**Totals**	**100.00**	

**Table:** (b)

Branch 2			Flower			Powder		
Element	Weight%	Atomic%	Element	Weight%	Atomic%	Element	Weight%	Atomic%
C K	39.52	50.12	C K	17.96	26.59	C K	42.10	50.31
O K	46.73	44.49	O K	46.35	51.52	O K	53.72	48.20
Si K	1.01	0.55	Mg K	0.39	0.29	Ca K	4.18	1.50
Ca K	12.74	4.84	Al K	1.67	1.10			
			Si K	31.10	19.70			
			Fe K	2.53	0.80			
**Totals**	**100.00**		**Totals**	**100.00**		**Totals**	**100.00**	

**Table 9:** Elemental analysis of *Anastatica hierochuntica* L. from the selected area.

Elemental Composition (%)																		
Extracts	O		C		Mg		Al		Si		Ca		K		Zn		Fe	
	Weight	Atomic	Weight	Atomic	Weight	Atomic	Weight	Atomic	Weight	Atomic	Weight	Atomic	Weight	Atomic	Weight	Atomic	Weight	Atomic
Branch 1	49.31	45.95	38.78	48.13	nd	nd	2.18	1.21	6.89	3.66	2.84	1.05	nd	nd	nd	nd	nd	nd
Branch 2	46.73	44.49	39.52	50.12	nd	nd	nd	nd	1.01	0.55	12.74	4.84	nd	nd	nd	nd	nd	nd
Stem	52.42	57.50	16.98	24.81	1.25	0.90	5.09	3.31	18.51	11.57	0.78	0.34	1.33	0.59	3.64	0.98	nd	nd
Stigma	42.73	42.63	35.19	46.77	0.85	0.56	3.61	2.13	7.19	4.09	6.11	2.43	1.19	0.49	nd	nd	3.14	0.90
Flower	46.35	51.52	17.96	26.59	0.39	0.29	1.67	1.10	31.10	19.70	nd	nd	nd	nd	nd	nd	2.53	0.80
Powder	53.72	48.20	42.10	50.31	nd	nd	nd	nd	nd	nd	4.18	1.50	nd	nd	nd	nd	nd	nd

nd: not detected.
